# Advances in the development of SUMO specific protease (SENP) inhibitors

**DOI:** 10.1016/j.csbj.2015.03.001

**Published:** 2015-03-24

**Authors:** Ashutosh Kumar, Kam Y.J. Zhang

**Affiliations:** Structural Bioinformatics Team, Division of Structural and Synthetic Biology, Center for Life Science Technologies, RIKEN, 1-7-22 Suehiro, Yokohama, Kanagawa 230-0045, Japan

**Keywords:** Sumoylation, SUMO, SENP, Prostate cancer, Virtual screening

## Abstract

Sumoylation is a reversible post-translational modification that involves the covalent attachment of small ubiquitin-like modifier (SUMO) proteins to their substrate proteins. Prior to their conjugation, SUMO proteins need to be proteolytically processed from its precursor form to mature or active form. SUMO specific proteases (SENPs) are cysteine proteases that cleave the pro or inactive form of SUMO at C-terminus using its hydrolase activity to expose two glycine residues. SENPs also catalyze the de-conjugation of SUMO proteins using their isopeptidase activity, which is crucial for recycling of SUMO from substrate proteins. SENPs are important for maintaining the balance between sumoylated and unsumoylated proteins required for normal cellular physiology. Several studies reported the overexpression of SENPs in disease conditions and highlighted their role in the development of various diseases, especially cancer. In this review, we will address the current biological understanding of various SENP isoforms and their role in the pathogenesis of different cancers and other diseases. We will then discuss the advances in the development of protein-based, peptidyl and small molecule inhibitors of various SENP isoforms. Finally, we will summarize successful examples of computational screening that allowed the identification of SENP inhibitors with therapeutic potential.

## 1 SUMO and sumoylation

**Fig. 1:**
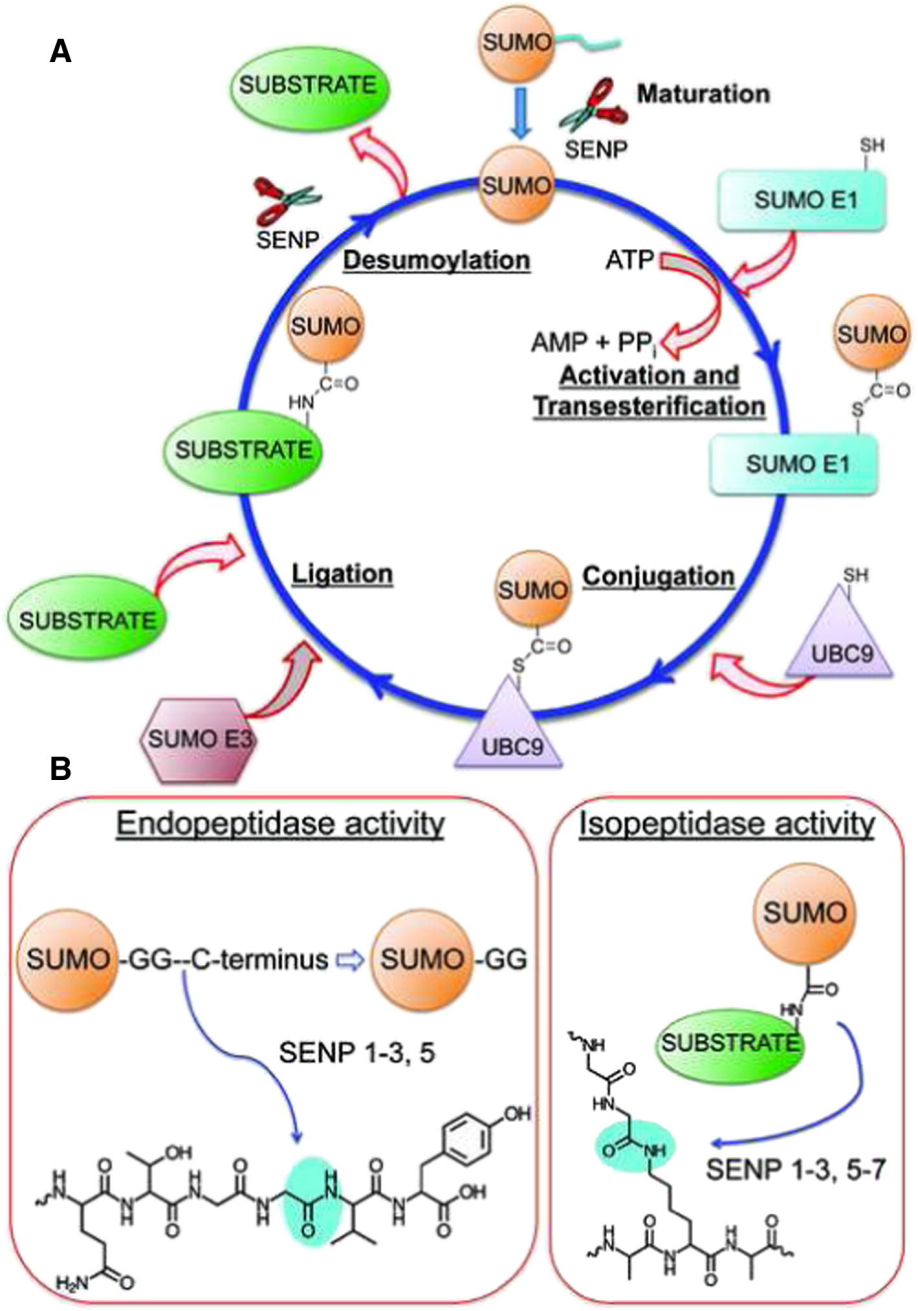
Sumoylation and desumoylation pathway. (A) In sumoylation, SUMO protein is covalently attached to lysine residues in target proteins via a sequential action of an activating enzyme E1, a conjugating enzyme E2, and a ligase E3. (B) SENPs possess endopeptidase activity to carry out proteolytic processing at the precursor SUMO C-terminus (SUMO-2 C-terminus is shown here) to expose two glycine residues. SENPs also possess isopeptidase activity to release conjugated SUMO from substrate proteins.

Sumoylation is a reversible process that involves the post-translational modification of target proteins with an approximately 10 kDa small ubiquitin-like modifier (SUMO) protein. Sumoylation is an important mechanism regulating the activities of various proteins involved in cellular processes like DNA replication and repair, chromosome packing and dynamics, genome integrity, nuclear transport, signal transduction and cell proliferation [1–5]. In sumoylation SUMO proteins are covalently attached to the ε-amino group of lysine residues in specific target proteins via an enzymatic cascade that requires a sequential action of an activating enzyme E1, a conjugating enzyme E2 and a ligase E3 [1–5] (Fig. 1A). The first step in the sumoylation pathway involves the conjugation of a SUMO protein to SUMO activating enzyme 1 (SUMO E1) that starts with the binding of ATP. The SUMO E1 subsequently catalyzes the adenylation of SUMO C-terminus at the expense of ATP to form SUMO-AMP intermediate. Following the formation of SUMO-AMP intermediate, the catalytic residue Cys173 on SUMO E1 and SUMO-AMP intermediate came close as the result of a large conformational change in SUMO E1 [6] which is followed by the transfer of SUMO to the catalytic residue Cys173 on SUMO E1 by the formation of a thioester bond between SUMO C-terminus and Cys173 [6–8]. The next step involves the subsequent transfer of SUMO protein from SUMO E1 to SUMO E2 (also known as Ubc9); again with the formation of a thioester linkage between the C-terminal glycine in SUMO protein and the catalytic Cys93 in SUMO E2. Ubc9 is the only known SUMO E2 enzyme, and its deletion abolishes SUMO conjugation [9–11]. In the last step, Ubc9 catalyzes the covalent attachment of the SUMO protein to the ε-amino group in a specific lysine residue of substrate proteins. SUMO E3 ligase increases the efficiency of this step by associating with both the substrate protein and Ubc9 [12–14].

## 2 SENP and SENP isoforms

Mammalian cells express four SUMO isoforms: SUMO-1, SUMO-2, SUMO-3 and SUMO-4. The mammalian SUMO-2 and SUMO-3 are much more similar to each other than to SUMO-1. SUMO-4 on the other hand is highly homologous with SUMO-2 and SUMO-3, but its expression is limited only to a few tissues and organs, mainly the kidney, lymph and spleen [15,16]. SUMO proteins are expressed as precursors and need to be proteolytically processed from its pro or inactive form to mature form [4,17–21]. Sentrin/SUMO-specific proteases (SENPs) cleave inactive or pro form of SUMO at the C-terminus via its hydrolase activity to expose two glycine residues and thereby generating active or mature SUMO (Fig. 1B) [4,17–21]. In addition to C-terminal proteolytic processing, SENPs also possess isopeptidase activity that is essential for the recycling of SUMO proteins [4,17–21]. These enzymes specifically cleave the isopeptide bond between the C-terminal glycine of SUMO and the substrate protein lysine thereby releasing the SUMO protein from its substrate (Fig. 1B).

Six SENP isoforms (SENP1, SENP2, SENP3, SENP5, SENP6 and SENP7) have been identified in mammals. These six SENPs can be divided into three sub-families based on their sequence homology, substrate specificity and subcellular localization as shown in Table 1 [4,17–21]. SENP1 and SENP2 constitute the first family and have broad specificity for SUMO-1/2/3. SENP3 and SENP5 form the second family, while the third family has SENP6 and SENP7 as its members. Apart from SENP1 and SENP2, all other SENP isoforms prefer SUMO-2/3 over SUMO-1 for deconjugation. The posttranslational modification of substrate proteins by SUMO-4 has not been observed due to SENP's inability to proteolytically process SUMO-4 precursor in vivo [16,22]. The maturation of precursor SUMO-4 seems to be inhibited by the presence of Pro90 residue in place of Gln in SUMO-1–3. Pro90 causes conformational constraint and makes the peptide bond to be cleaved inaccessible to the narrow active site of SENP [22,23]. A P50Q single amino acid mutation made the precursor SUMO-4 amenable to SENP2 processing while another additional mutation G63D made it a highly efficient SENP2 substrate [24]. As for their distribution, SENP1, SENP6 and SENP7 are localized in the nucleoplasm while SENP3 and SENP5 are confined to the nucleolus. Although SENP2 is compartmentalized in the nuclear pore complex, however, along with SENP1 it possesses nuclear export signal to facilitate its shuttling in and out of the nucleus. While all six isoforms possess isopeptidase activity, only SENP1, SENP2 and SENP5 can carry out proteolytic processing of precursor SUMOs.

The N-terminal regions of all six SENP isoforms are more or less unrelated while a conserved cysteine protease catalytic domain was observed at the C-terminus [21]. The catalytic cysteine protease domain, which is the most studied region, is approximately 250 amino acid residues long and controls the specificity and function of SENP isoforms. The N-terminal region is poorly conserved and thought to regulate the localization of SENP isoforms [21]. The three-dimensional (3D) structural information is available only for the catalytic domains of SENP1, SENP2 and SENP7 (Table 1). The crystal structures are either in apo form or in complex with SUMO proteins and a substrate RanGAP1. The catalytic domain crystal structures of SENP1, SENP2 and SENP7 are very similar [25–30]. The catalytic site is comprised of the typical catalytic triad (Cys-His-Asp) (Cys603, His533 and Asp550 for SENP1; Cys-548, His-478, and Asp-495 for SENP2; His-794, Cys-926 and Asp-873 for SENP7) analogous to other cysteine proteases. The catalytic triad is very important for precursor processing and deconjugation activities of SENPs and mutation of the catalytic triad residues abolishes the functional activity [26]. SUMO proteins enter the catalytic site via a narrow tunnel lined by Trp residues, which are essential for the accurate positioning of Gly–Gly motif and sessile bond. It has been revealed that the sessile bond is oriented in a cis-configuration that creates a kink in SUMO precursor tails and isopeptide linkage of sumoylated substrate proteins. These types of cis-peptide bonds are not stable and promote cleavage [25].

## 3 Role of SENPs in the development of various diseases

SENP enzymes play a critical role in maintaining normal cellular physiology by preserving the balance between sumoylated and unsumoylated proteins. Knockout studies in mice have shown that the absence of SENP1 or SENP2 is embryonically lethal [31,32]. However in diseased states, this balance between SUMO modified and non-modified proteins is disrupted due to the altered expression of SENPs. Several studies implicated the role of various SENP isoforms in the development of various diseases, including prostate cancer [31,33–35], thyroid cancer [36], colon cancer [37], pancreatic cancer [38], atherosclerosis [39] and heart diseases [40]. Especially, numerous studies indicated the role of SENPs in prostate cancer development. Androgen receptor (AR) is the most important protein in the development and progression of prostate cancer. SENP1 and SENP2 are involved in the desumoylation of AR and studies have shown that the overexpression of SENP1 increases AR transcriptional activity [41,42]. In prostate cancer, hypoxia-inducible factor 1α (HIF-1α) plays a critical role in regulating the expression of various genes required for enhanced oxygen availability in hypoxic tissue environments [43,44]. The sumoylation of HIF-1α has been reported to have varied outcomes by several groups [31,45–47]. Polycomb chromobox 4 (CBX4) and RWD-containing sumoylation enhancer (RSUME) increased the HIF-1α sumoylation by promoting the transcriptional activity of HIF-1α during hypoxia [47,48]. Moreover, it is proposed that the stability and/or transcriptional activity of HIF-1α have been modulated via different sumoylation patterns by CBX4 and the protein inhibitor of activated STAT protein gamma (PIASγ) [48]. Further, it has been shown that SENP1 is essential for HIF-1α stability and consequently the regulation of hypoxic response [31]. Moreover, the absence of SENP1 resulted in active sumoylation of HIF-1α and ubiquitin dependent degradation [31]. In another study, Bawa-Khalfe et al. [33] demonstrated the high correlation between HIF-1α and SENP1 expression, indicating SENP1's role in prostate carcinogenesis. Furthermore, the overexpression of SENP1 has been observed in more than half of the studied samples of prostate cancer and prostatic intraepithelial neoplasia lesions [33,35]. Analyzing more than 150 specimens of prostate cancer, Wang et al. also demonstrated the correlation between SENP1 expression and prostate cancer aggressiveness and recurrence [49]. Other evidences for the role of SENP1 in prostate cancer tumorigenesis include the increase in androgen receptor activity [42] and c-Jun dependent transcription [50]. Xu et al. [37] demonstrated the regulation of in vivo and in vitro growth of colon cancer cells by SENP1. They reported that SENP1 overexpressed in most of the colon cancer tissues and its silencing arrested the cell growth in nude mice and the colony formation in a colon cancer cell line DLD-1. Ma et al. [38] described the overexpression of SENP1 in pancreatic ductal adenocarcinoma in comparison with normal tissue. They further showed that knockdown of SENP1 using siRNA inhibited the growth of pancreatic ductal adenocarcinoma.

Although SENP1 is the most studied isoform, the role of other SENP isoforms in pathogenesis has also been reported. SENP2 associates with MDM2 and regulates its sumoylation levels, which is important for its binding with tumor suppressor p53 [51,52]. On the other hand, SENP2 regulates the stability of β-catenin via a WW domain-containing oxidoreductase (WWOX) and thereby regulates the growth of hepatocellular carcinoma cells [53,54]. SENP2 also contributes to the atherosclerotic plaque formation by regulating the levels of p53 and extracellular signal-regulated kinase-5 (ERK5) under disturbed flow conditions [39]. Furthermore, the overexpression of SENP2 is also associated with congenital heart defects and cardiac dysfunction in murine hearts [40]. Other SENPs, especially SENP3 and SENP5, are also therapeutically relevant, as their elevated expression levels have been observed in prostate, ovarian, lung, colon, oral squamous cell carcinomas and osteosarcoma [55–58]. SENP3 is involved in increasing the transcriptional activity of HIF-1α through the desumoylation of a co-regulator p300 [59]. In another study utilizing gene expression datasets from 1363 patients, Cashman et al. [60] demonstrated the correlation between low expression of SENP5 and breast cancer patient survival.

## 4 Identification of SENP inhibitors

Since SENPs play a critical role in the development of various diseases including cancer, atherosclerosis and heart diseases, designing and developing novel inhibitors are of paramount importance. Hence, there is a growing interest among researchers to discover selective inhibitors of SENP isoforms. Several groups focused on the development of inhibitors of various SENPs useful as chemical tools for studying biological roles of sumoylation and desumoylation as well as for exploring the therapeutic potential of SENPs.

**Fig. 2:**
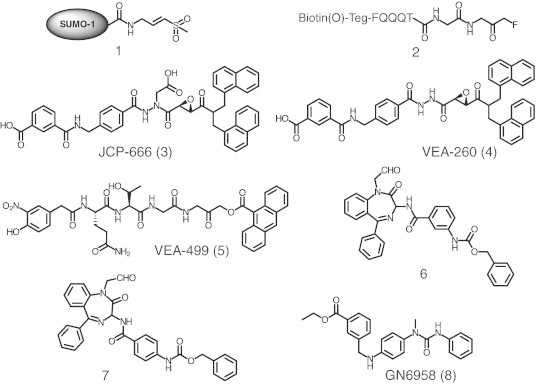
A few representative protein-based, peptidyl and small molecule inhibitors of SENPs.

One of the earliest SENP inhibitor development strategies made use of the full or truncated form of SUMO carrying an electrophilic trap or “warhead” at the C-terminal glycine. In one study, an intein-based method was employed to equip SUMO-1 and other ubiquitin like proteins with a vinyl sulfone (VS) as an electrophilic trap [61]. These protein-based probes reacted covalently with SENP2 (**1** in Fig. 2) and other activating, conjugating and deconjugating enzymes through Michael addition of the catalytic cysteine thiol group with VS moiety. Pre-incubation of SENP2 with n-ethylmaleimide (NEM), an alkylating agent, prevented the formation of SUMO-1-VS-SENP2 conjugate and confirmed that cysteine is required for catalysis [61]. A similar strategy was used by Borodovsky et al. [62] to synthesize several peptides with various portions of the C-terminus of ubiquitin-like modifiers Nedd8, SUMO1, FAT10, Fau, and APG12 equipped with a VS electrophilic trap. A dose-dependent labeling of at least one cell lysate protein by SUMO1-peptide-VS was shown in this study. Dobrota et al. [63] also reported the synthesis of a peptidyl active site probe (**2** in Fig. 2) for SENP1 and SENP2 using a similar approach. This compound contains an electrophilic trap glycine fluoromethylketone at the C-terminus of a seven-residue SENP specific peptide (FQQQTGG). In 2011, Ponder et al. [64] reported a small molecule inhibitor (JCP-666, **3** in Fig. 2) of *Plasmodium falciparum* SENP1 (*Pf*SENP1) by screening a focused library of cysteine protease inhibitors. JCP-666 harbors a reactive aza-epoxide linked to a non-natural peptide backbone and displayed an IC_50_ of 17.9 μM for *Pf*SENP1. A more stable synthetic analog (VEA-260, **4** in Fig. 2) without the aspartic acid side-chain on the aza-epoxide scaffold showed similar potency against *Pf*SENP1 (16.2 μM). It is interesting to note that both compounds also exhibited excellent potency against human SENP1 and SENP2 [64]. Compound **3** displayed IC_50_ of 9.0 and 4.7 μM for human SENP1 and SENP2 respectively, while compound **4** showed slightly better activity of 7.1 and 3.7 μM respectively for human SENP1 and SENP2. Using compound **4** as the starting point, Albrow et al. [65] synthesized 16 compounds. The inhibitory potency of these compounds was evaluated against human SENP1, 2, 5, 6 and 7. However, all the synthesized compounds were either less or equipotent as the parent compound. Moreover, these aza-epoxide based active site probes demonstrated high background labeling when used in complex proteomes indicating their non-specific nature [65]. In the same report, Albrow et al. [65] synthesized another series (11 compounds) of human SENP inhibitors based on the compound **4** scaffold and natural SUMO/ubiquitin amino acid sequence accommodating the acyloxymethyl ketone (AOMK) reactive group. Bioactivity evaluations and subsequent IC_50_ determination of more potent compounds revealed that VEA-499 (**5** in Fig. 2) was the most potent inhibitor with IC_50_ values of 3.6 and 0.25 μM for human SENP1 and SENP2 respectively [65]. Furthermore, AOMK based inhibitors were also good active site probes as they exhibited highly specific binding in complex proteomes.

In light of the poor pharmacokinetic properties of peptidyl inhibitors, Qiao et al. [66] designed and synthesized a series of benzodiazepine based SENP1 inhibitors. SENP1 activity was evaluated using SUMO-CHOP reporter fluorescence assay [67]. Two most potent compounds (compounds **6** and **7** in Fig. 2) displayed IC_50_ of 15.5 and 9.2 μM. Compounds **6** and **7** also inhibited cancer cell growth in vitro with IC_50_ values of 13.0 and 35.7 μM respectively. In another attempt to develop SENP1 inhibitors as potential anti-cancer agents, Uno et al. [68] designed and synthesized 1-[4-(*N*-benzylamino)phenyl]-3-phenylurea derivatives based on a potent HIF-1α inhibitor. The most potent compound (GN6958, **8** in Fig. 2) displayed selective SENP1 inhibition with an IC_50_ of 29.6 μM. Like the parent compound, compound **8** also suppressed HIF-1α without affecting tubulin expression [68]. Another study reported the down-regulation of SENP1 expression at both mRNA and protein levels by the natural product triptolide and thereby enhancing sumoylation in prostate cancer cells [69]. However, the actual mechanism of SENP1 downregulation is not known. Recently utilizing virtual screening approach, several groups reported inhibitors of various SENP isoforms. These include 2-(4-chlorophenyl)-2-oxoethyl 4-benzamidobenzoate analogs [70], non-covalent SENP inhibitors containing a sulfonyl-benzene group [71], 1,2,5-oxadiazoles [72] and a cell permeable SENP specific inhibitor [73]. The identification and biological properties of these inhibitors are summarized in Table 2 and described in detail below.

## 5 Computational approaches in the identification of SENP inhibitors

**Fig. 3:**
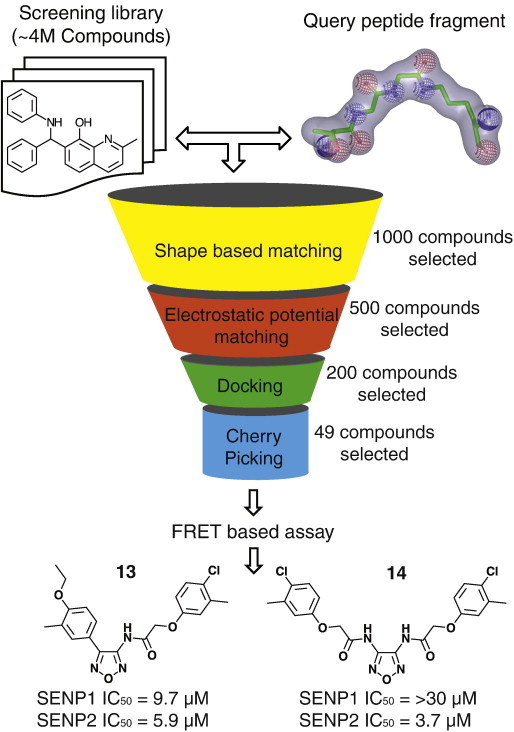
An outline of the discovery of 1,2,5-oxadiazoles as novel inhibitors of SENP1 and SENP2 utilizing a hierarchical virtual screening approach.

In the last two decades, computational approaches have played a noteworthy role in the identification and optimization of small molecule inhibitors of proteins of therapeutic interests [74,75]. Taking advantages of virtual screening over conventional high-throughput screening, several groups employed virtual screening in combination with biological assay to identify small molecule inhibitors of various SENP isoforms [70–73]. Chen et al. [70] reported SENP1 inhibitors which were identified by virtual screening for the first time. They docked SPECS library of about 180,000 compounds to SENP1 from the SENP1–SUMO2–RanGAP1 crystal structure. Thirty-eight compounds were selected and purchased from the top scoring 100 compounds. Assessment of bioactivity utilizing fluorescence based assay resulted in the identification of compound **9** (Table 2) with an IC_50_ of 2.39 μM. Docking predicted binding mode of compound **9** was further used to guide the design and synthesis of 2-(4-chlorophenyl)-2-oxoethyl 4-benzamidobenzoate analogs. However, no significant improvement in the activity (best IC_50_ of 1.08 μM for compound **10**) over the parent compound was observed [70]. Madu et al. [71] virtually screened a 250,000 compound National Cancer Institute library using Glide program to obtain 40 compounds for the evaluation of SENP1 and SENP2 inhibitory activities. A gel-based assay that quantifies the maturation of SUMO-1 and SUMO-2 precursors was used to investigate inhibitory effects. A luciferase based coupled bioluminescent assay was also used to determine the inhibition of SENP7 along with SENP1 and SENP2. Bioassay of initial hits and subsequent analog search revealed compounds with a novel chemotype (compounds **11** and **12** in Table 2) that do not covalently modify the catalytic cysteine. The non-competitive inhibitory nature of these compounds was confirmed using nuclear magnetic resonance (NMR) and quantitative enzyme kinetic experiments. Employing a combination of hierarchical virtual screening and quantitative FRET based assay, Kumar et al. reported 1,2,5-oxadiazoles as novel inhibitors of SENP1 and SENP2 [72]. An overview of the virtual screening protocol used by Kumar et al. is presented in Fig. 3. In summary, an ~ 4 M compound library was screened for small molecules that have similar shape and electrostatic properties with the conjugate of SUMO-1 C-terminal residues and substrate lysine. Docking and diversity based selection outlined a set of 49 compounds to be tested for SENP1 and SENP2 inhibitory activities. FRET based assay and subsequent analog search revealed two 1,2,5-oxadiazole core containing scaffolds as a novel class of SENP1 and SENP2 inhibitors. Identified inhibitors were specific to SENP and no detectable inhibition on other proteases, such as papain and trypsin, was observed. Wen et al. [73] also utilized hierarchical virtual screening to identify a novel cell-permeable inhibitor of SENP1. They docked about 100,000 drug-like compounds from SPECS database in a stepwise manner using DOCK and AutoDock program. Cherry picking from the top scoring 500 compounds resulted in the selection of 117 compounds for purchase and evaluation of SENP1 inhibitory activity using a SUMO-CHOP reporter assay [67]. The most potent compound (compound **15** in Table 2) displayed an IC_50_ of 1.29 μM for SENP1. Compound **15** was also re-evaluated using in vitro gel-based SENP activity assay to confirm the inhibitory activity that revealed the inhibition SENP1-mediated cleavage of ΔRanGAP1-SUMO-2. Further biological assay revealed that compound **15** is a relatively specific SENP inhibitor and had little or no effect on the activity of proteasome and other cysteine proteases such as cathepsin B and cathepsin D. Molecular docking was employed to propose a mechanism of inhibition that showed that compound **15** binds in a tunnel preventing the binding of SUMO-1 to SENP1. Computational approaches have been also used indirectly to facilitate SENP inhibitor discovery efforts from various groups. Adopting molecular dynamics simulation and quantum mechanics/molecular mechanics calculations, Shi et al. [76] deciphered the catalytic mechanism of SENP desumoylation. They identified critical residues in SENP1 that might be useful in its inhibitor discovery. In another study, Kumar et al. demonstrated the inhibition of SENP:SUMO protein–protein interaction as a viable alternative strategy to target the enzymatic site [77].

## 6 Concluding remarks

As discussed in this review, increasing evidence suggests the role of various SENP isoforms in the development of a number of diseases, especially prostate cancer. Several studies indicated that inhibition of SENPs might be a good approach to combat various cancers. In recent years, considerable progress has been made towards the identification of small molecule inhibitors of various SENP isoforms. However, most of the developed inhibitors are only suitable as probe molecules to study the biological mechanism of various SENPs. The therapeutic potential of most of the presently identified inhibitors is limited, as they possess reactive chemical functionalities to facilitate covalent binding with the active site cysteine. Moreover, none of the identified chemical classes is isoform specific. Similar biological mechanisms, structural features and chemistry behind the peptide cleavage make it difficult to identify isoform selective SENP inhibitors. Computational approaches like molecular docking have been successfully employed in recent years to identify chemical scaffolds that are much more amenable for chemical optimization needed for lead development. However, many more drug-like chemical scaffolds need to be identified along with their structural description of binding to exploit the therapeutic potential of SENP inhibitors.

## Figures and Tables

**Table 1:** Biological properties and structural information of SENP isoforms.

SENP isoform	Specificity	Subcellular localization	Enzymatic activity	Amino acid residues	Structural information
SENP1	SUMO-1/2/3	Nucleoplasm	Precursor processing, isopeptidase	643 Catalytic domain (419–643)	Apo (**2IYC**, **2XPH**, **2XRE**, **2CKG**) With SUMO-1 (**2IY1**, **2G4D**) With SUMO-2 (**2IYD**, **2CKH**) With SUMO-1 and substrate RanGAP1 (**2IY0**)
SENP2	SUMO-1/2/3	Nuclear pore	Precursor processing, isopeptidase	589 Catalytic domain (365–589)	Apo (**1TH0**) With SUMO-1 (**1TGZ**) With preSUMO-2 (**2IO0**, **3ZO5**) With preSUMO-3 (**2IO1**) With SUMO-1 and substrate RanGAP1 (**2IO2**) With SUMO-2 and substrate RanGAP1 (**2IO3**)
SENP3	SUMO-2/3	Nucleolus	Isopeptidase	574 Catalytic domain (353–574)	Not available
SENP5	SUMO-2/3	Nucleolus	Precursor processing, isopeptidase	755 Catalytic domain (567–755)	Not available
SENP6	SUMO-2/3	Nucleoplasm	Isopeptidase	1112 Catalytic domain (637–1112)	Not available
SENP7	SUMO-2/3	Nucleoplasm	Isopeptidase	984 Catalytic domain (662–984)	Apo (**3EAY**)

**Table 2:** Overview of SENP inhibitors identified using virtual screening.

SENP target	Structure of representative compounds	Activity of most potent compounds	Virtual screening method used	Reference
SENP1		Compound **9** IC_50_ = 2.38 μM Compound **10** IC_50_ = 1.08 μM	Molecular docking of 180,000 compound library using Glide program.	Chen et al. [70]
SENP1, SENP2, SENP7		Compound **11** SENP1 IC_50_ = 5.9 μM SENP2 IC_50_ = 2.9 μM SENP7 IC_50_ = 3.5 μM Compound **12** SENP1 IC_50_ = 2.1 μM SENP2 IC_50_ = 2.0 μM SENP2 IC_50_ = 2.7 μM	Molecular docking of 250,000 compound library using Glide program.	Madu et al. [71]
SENP1, SENP2		Compound **13** SENP1 IC_50_ = 9.7 μM SENP2 IC_50_ = 5.9 μM Compound **14** SENP1 IC_50_ = > 30 μM SENP2 IC_50_ = 3.7 μM	Hierarchical virtual screening of ~ 4 million compound library by shape and electrostatic similarity search using ROCS and EON program. Molecular docking using Glide program prioritized hits for bioassay.	Kumar et al. [72]
SENP1		Compound **15** IC_50_ = 1.29 μM	Molecular docking of 100,000 compound library using Dock and Autodock program.	Wen et al. [73]
